# Comparing the Climatic and Landscape Risk Factors for Lyme Disease Cases in the Upper Midwest and Northeast United States

**DOI:** 10.3390/ijerph17051548

**Published:** 2020-02-28

**Authors:** Yuting Dong, Zheng Huang, Yong Zhang, Yingying X.G. Wang, Yang La

**Affiliations:** 1 College of Life Sciences, Nanjing Normal University, Nanjing 210046, China; 2 College of Biology and the Environment, Nanjing Forestry University, Nanjing 210037, China; 3 Department of Biological and Environmental Science, University of Jyväskylä, FI-40014 Jyväskylä, Finland; 4 Medical College, Tibet University, Lhasa 850000, China

**Keywords:** lyme disease, *Borrelia burgdorferi*, forest fragmentation, climate

## Abstract

Lyme disease, recognized as one of the most important vector-borne diseases worldwide, has been increasing in incidence and spatial extend in United States. In the Northeast and Upper Midwest, Lyme disease is transmitted by *Ixodes scapularis*. Currently, many studies have been conducted to identify factors influencing Lyme disease risk in the Northeast, however, relatively few studies focused on the Upper Midwest. In this study, we explored and compared the climatic and landscape factors that shape the spatial patterns of human Lyme cases in these two regions, using the generalized linear mixed models. Our results showed that climatic variables generally had opposite correlations with Lyme disease risk, while landscape factors usually had similar effects in these two regions. High precipitation and low temperature were correlated with high Lyme disease risk in the Upper Midwest, while with low Lyme disease risk in the Northeast. In both regions, size and fragmentation related factors of residential area showed positive correlations with Lyme disease risk. Deciduous forests and evergreen forests had opposite effects on Lyme disease risk, but the effects were consistent between two regions. In general, this study provides new insight into understanding the differences of risk factors of human Lyme disease risk in these two regions.

## 1. Introduction

Lyme disease, caused by spirochete *Borrelia burgdorferi* sensu stricto (*B. burgdorferi* hereafter), is recognized as one of the most important vector-borne diseases in United States [1,2]. Since Lyme disease was first reported in Connecticut in 1975 [3,4], it has been increasing in incidence and spatial extend in United States [5,6]. Now, Lyme disease is endemic in the Northeast, Upper Midwest and West Coast [1]. In the Northeast and Upper Midwest, Lyme disease is vectored by deer ticks (*Ixodes scapularis*), which maintain *B. burgdorferi* in a horizontal transmission cycle between ticks and multiple vertebrate hosts [7]. Disease ecologists have made great efforts to understand the transmission processes of *B. burgdorferi* and identified many biotic and abiotic risk factors that attribute to Lyme disease expansion and spread in United States [1,3,8], and these efforts have yielded a wide range of control strategies. However, the number of Lyme cases have steadily increased, with about 30,000 cases of Lyme disease (according to CDC reports) occurred annually now in United States [9]. As currently no human vaccines are available [10], a better understanding of the epidemiology and risk factors of Lyme disease is still needed.

As the process of Lyme disease spread involves hosts, vectors and pathogens, any factors that can potentially influence their survivals, distributions and movements may affect the risk of disease transmission [11,12]. Previous studies have identified many climatic and landscape factors that may attribute to Lyme disease risk [1,13,14,15,16]. For climatic factors, laboratory studies had shown that ticks are highly vulnerable to desiccation and generally had high mortality in conditions with low humidity and high temperature [17,18]. Thus, temperature and humidity may affect Lyme disease risk indirectly through the impacts on tick survivals and population dynamics [19,20,21]. For example, when investigating Lyme incidence in seven northeastern states, Subac found a positive relationship between disease incidence and the June moisture index in previous years [22]. This result might be explained by a later field work study which showed that heavy precipitation in late spring or early summer precipitation was the most favorable climatic factor for tick survival in the Northeast [21]. Besides precipitation and humidity, temperature has also been correlated to Lyme disease risk. A recent study exploring the county-level Lyme spread across the United States found that the mean temperature was negatively correlated with Lyme disease spread [6], which was consistent to a previous study which also showed a negative correlation between the county-level Lyme incidence and the maximum annual temperature in the Northeast [3].

For landscape factors, a previous review had suggested that the presence of forest was consistently associated with increased Lyme disease risk [1]. Besides, forest habitat configurations can also be important in affecting Lyme disease risk due to its impacts on host movements and distributions, as well as the contact rates between human and ticks [23,24,25,26]. Human activity like urbanization induced fragmentation, increasing the amount of edge habitats between residential development and forests [3,27]. These edge habitats serve as preferred habitats for many host species of ticks, particularly the white-tailed deer that is the main host for adult ticks [28], and thus can increase the entomological risk of Lyme disease [29]. Forest fragmentation may also increase the contact rates between human population and ticks, which can elevate human exposure to Lyme disease [30]. However, there is also a different mechanism, suggesting that the spread of pathogens and tick vectors may be slowed down in fragmented patches due to the restriction on host movements [31].

When retrospecting studies on the risk factors of Lyme disease in the United States, we may find that relatively fewer studies focused on the Upper Midwest, comparing to the Northeast. It has been suggested that Lyme disease in these two regions originated from different places (Connecticut for the Northeast, and Wisconsin for the Upper Midwest) [4]. Besides, the seasonality in tick feeding also showed some differences, though *B. burgdorferi* is typically transmitted by the same tick species *I. scapularis* in these two regions. In the Northeast, nymphs feed predominantly during May and July, and larvae mainly take their bloodmeals from June to September, while the seasonal timing of larval and nymphal feeding coincide in the Upper Midwest [32]. This seasonal synchrony in nymphal and larval feeding may make the Lyme dynamics and risk factors different to those in the Northeast. In this study, we explore the climatic and landscape factors that influence the spatial patterns of Lyme cases and compare the risk factors in the Northeast and Upper Midwest United States. Our results suggested that climatic variables generally showed opposite correlations with Lyme disease risk, while landscape factors usually had similar effects in these two regions.

## 2. Materials and Methods

### 2.1. Lyme Disease Data

**Figure 1:**
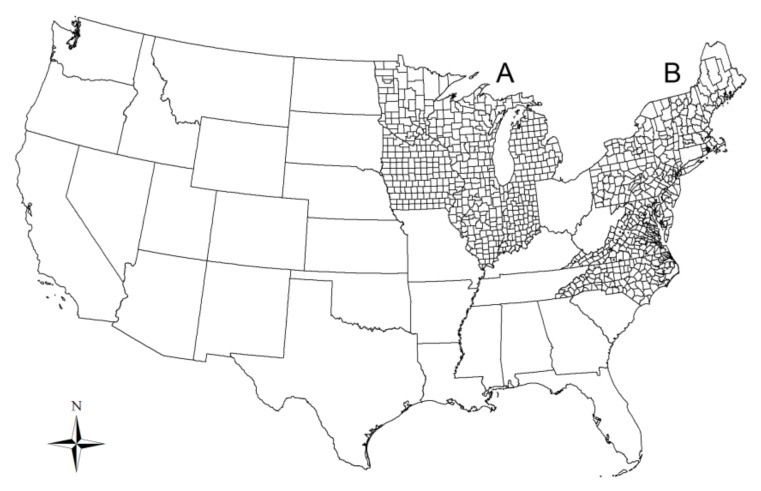
Map of study area. A—the Upper Midwest; B—the Northeast of United States.

The study area (Figure 1) includes 13 states in the Northeast (Connecticut, Delaware, Maine, Maryland, Massachusetts, New Hampshire, New Jersey, New York, North Carolina, Pennsylvania, Rhode Island, Vermont, and Virginia; not all of these states are considered to be in the Northeast, but here we follow a previous study [3], including all 13 states due to their geographical contiguity and high Lyme incidence) and six states in the Upper Midwest (Illinois, Indiana, Iowa, Michigan, Minnesota, Wisconsin) of United States. The annual number of human Lyme disease cases for each county during 2012–2016 were obtained from the Centers for Disease Control and Prevention (CDC; http://www.cdc.gov/lyme/stats/). According to a previous study [11], we limited our study area to those counties with established or reported *I. scapularis* populations.

### 2.2. Data of Predictors

For each county in each year, we calculated the mean temperature (MeanTem), maximum temperature (MaxTem) and mean precipitation (Pre) of each season (spring, summer, autumn, and winter) in previous year (Table 1), based on the Climate Research Unit (CRU) datasets [33], a time-series dataset that yields month-by-month variations in climate. The processing of climatic data was carried out in ArcGIS 10.2.2.

Land cover data of 2013 was accessed from the National Land Cover Database (NLCD) [34]. Following a previous study [3], we focused on seven particular land cover classes: deciduous forest (class 41), evergreen forest (class 42), mixed forest (class 43), developed-open space (class 21), developed-low intensity space (class 22), developed-medium intensity space (class 23), and developed-high intensity space (class 24). For each county, we then derived several landscape indicators for each land cover class (Table 1), including CA (total area of a specific land cover class), PLAND (percentage of a land cover respect to the total county area), TE (total edge length), ED (edge density, total edge length divided by the total county area). Following a previous study [5], we also include, in addition to climatic and landscape predictors, the distance to the origin areas of Lyme disease (Connecticut for Northeast and Wisconsin for Upper Midwest). The processing of landscape data was carried out in ArcGIS 10.2.2 and Fragstats 4.2.

### 2.3. Statistical Analyses

Following previous studies [3,5,35], we applied generalized linear mixed models (GLMM) with negative binomial regression to investigate the relationships between Lyme disease cases and predictors, as negative binomial regression allows for the overdispersion that was commonly encountered in reported cases of Lyme disease [29,36]. We included state and year as random factors to control for the variations between years and states. Before performing GLMMs, we scaled all predictor variables to have a mean of zero and a standard deviation of one.

With GLMMs, we first conducted univariate regression analyses to test the association of each predictor with Lyme disease risk. Predictors with a *p*-value < 0.05 were identified as potential risk factors which were used to conduct model averaging. Before performing model averaging, we checked for the multicollinearity by examining the correlation coefficients (*r*) between potential risk factors. For highly correlated factors (*r* > 0.7) [37], we only included the variable with the smaller *p*-value in model averaging. After removing highly correlated predictors, we constructed a full model with all remained potential risk factors. Based on the changed Akaike information criterion (AICc) values [38], we then ranked the candidate models and considered the models within ΔAICc < 2 as competing models, which were used to average the regression coefficient of each predictor variable. For both univariate analyses and model averaging analyses, the county area (AREA) was retained in the model to control for the effect of area size. All statistical analyses were conducted in RStudio^®^version 1.1.463 (RStudio, Inc., Boston, MA, USA) with *lme4* [39] and *MuMIn* [40] packages.

## 3. Results

### 3.1. Univariate Regression Analyses

Our results from univariate analyses (Table 2) showed that the distance to original disease area (Dist_O) had a negative correlation with Lyme cases in both the Northeast and Upper Midwest. The mean summer precipitation in previous year (Pre_2) was positively correlated with Lyme cases in Upper Midwest, while the mean autumn precipitation (Pre_3) was negatively correlated with Lyme disease risk in Northeast. The seasonal maximum temperature in previous year generally had a better predictive power than the seasonal mean temperature. The maximum temperature generally had negative effects on Lyme disease risk in Upper Midwest, while had positive effects in Northeast.

For landscape predictors (Table 2), all four indicators (CA, PLAND, TE, ED) related to the developed area (land cover class: 41,42,43,44) generally had positive effects on Lyme disease risk in both the Upper Midwest and Northeast. The percentage of deciduous forest (PLAND_41) showed negative correlation with Lyme disease risk in the Upper Midwest, while the total edge length (TE_41) and edge density (ED_41) showed positive correlations in the Northeast. For evergreen forest, all four indicators had negative effects on Lyme disease risk in both regions. For mixed forest, the total forest area (CA_43), the percentage of forest area (PLAND_43) and the total edge length (TE_43) were negatively associated with Lyme disease risk in the Upper Midwest, while only the CA_43 had a significant negative effect in the Northeast.

### 3.2. Model Averaging Analyses

The results of model averaging (Table 3) showed that the distance to original disease area (Dist_O) had a negative correlation with Lyme cases in both the Northeast and Upper Midwest. Besides of Dist_O, the total edge length (TE_21), the edge density of open space developed area (ED_21), and the percentage of deciduous forests (PLAND_41) were positively associated with Lyme cases in the Upper Midwest. In the Northeast, the total edge length of low intensity developed area (TE_22), the edge density of deciduous forests (ED_41) and the percentage of high intensity developed area (PLAND_24) had positive effects on Lyme disease risk; while the percentage of evergreen forests (PLAND_42) have a negative effect.

## 4. Discussion

In this study, we explored the correlations of climatic and landscape factors with the Lyme cases at county level in the Northeast and Upper Midwest United States. The results from univariate analyses suggested that the landscape factors related to developed area and forests generally had similar effects on Lyme disease risk in the two regions. In contrast, climatic factors generally showed opposite relationships with Lyme disease risk in the two regions. The results from model averaging analyses in two regions only identified several but quite different risk factors. As many climatic and landscape factors were highly correlated with each other, the significant effect of a specific factor in multiple models might also be caused by other highly correlated factors. Therefore, we here focus more on discussing the results from univariate analyses.

In both regions, the seasonal mean maximum temperature in previous year were better than the mean temperature in previous year in explaining the spatial patterns of Lyme cases. Increasing the mean maximum temperature in previous year was associated with a decrease in the number of Lyme cases in Upper Midwest, while associated with an increase in Lyme disease risk in Northeast. The precipitation in previous summer was positively correlated with Lyme disease risk in Upper Midwest, while the precipitation in previous autumn showed a negative association in Northeast. The results from the Upper Midwest seems consistent to the expectation that low humidity and high temperatures could regulate tick abundance [21,22]. In contrast, the results from the Northeast conflicted with this expectation, but consistent with a previous study which also suggested a tick abundance when there was a high temperature at ground level [41]. These results confirmed the conclusion from a previous study which suggest that the effects of weather variables can vary considerably among different regions [42].

In contrast to climatic factors, most landscape factors showed similar effects on Lyme disease risk in the Northeast and Upper Midwest. Both the area size related indices (CA and PLAND) and fragmentation indices (TE and ED) of developed area (land cover class: 21–24) showed very strong positive correlations with Lyme disease risk (as seen in Table 2). As these indices were generally positively correlated with each other, we could not draw the conclusion which factors had true causal effects on Lyme disease risk. However, we found that in both regions, the multiple regression models included the fragmentation related indices of developed area (TE22 for Northeast; TE21 and ED21 for Upper Midwest; see Table 3), which might indicate that Lyme disease risk generally increased in fragmented developed area. These results were consistent to a previous study [3]. According to the NLCD 2013 classification, the open developed area (land cover class 21) and the low intensity developed area (class 22) are most likely single family housing units. The fragmentation of these types of land covers indicated a high chance of the occurrence of surrounding forests or herbaceous cover. Therefore, the contact rates between human and ticks might be enhanced in these areas [3]. Besides, edge habitats of residential area usually can provide more food resources for white tailed deer, the major host for adult ticks, increasing tick abundance [29]. Both of these two mechanisms could result in a higher Lyme disease risk in fragmented residential habitats.

The fragmentation of deciduous forests generally increases the number of Lyme cases (see Table 2). Previous studies have proposed that tick abundance is generally higher in fragmented deciduous forests, as forest fragmentations may provide ideal habitats for many reservoir hosts of ticks [1,30]. In fact, it had been shown that the entomological risk of Lyme disease risk was usually higher in small forest fragments due to the high abundance of white-footed mouse [29,30,43]. Moreover, edges in fragmented forests might be utilized more frequently by humans, resulting in higher contact rates between human and infected ticks [23]. After controlling for other factors in multiple regression models, the percentage of area of deciduous forests (PLAND 41) also had a positive effect in the Upper Midwest, consistent with many previous studies that had demonstrated the important role of forest cover in determining Lyme disease risk at landscape level. These studies suggested that more forest generally means more habitats for hosts, providing the blood meals for ticks, and thus the density of infected questing ticks [8,12,28]. In contrast to deciduous forests, the number of Lyme cases was lower in evergreen forests (class 42) in both regions. These results were also consistent to a previous study that suggested evergreen forests were located in mountainous areas, poor environments for ticks regarding to temperature and precipitation [3].

We must admit that the Lyme case number obtained from CDC might be an underestimate of actual human cases [1]. Particularly, different states may apply different approaches to gather case data. Including state as a random effect in our analyses was able to control for, to some extent, the differences in surveillance way among states.

## 5. Conclusions

In this study, we explored and compared the roles of climatic and landscape factors in shaping the spatial patterns of Lyme cases in the Upper Midwest and Northeast United States. Our results showed that climatic variables generally showed opposite correlations with Lyme disease risk, while landscape factors usually had similar effects in these two regions. High Lyme disease risk was correlated with high precipitation and low temperature in the Upper Midwest, while with low precipitation and high temperature in the Northeast. In both regions, area size related and fragmentation related indices of developed area showed strong positive correlations with Lyme disease risk. Deciduous forests and evergreen forests had opposite effects on Lyme disease risk, but the effects were consistent between two regions. Our study is the first study, to our knowledge, comparing the risk factors for Lyme disease in the Upper Midwest and the Northeast, and thus may provide new insight into understanding the differences of risk factors of Lyme disease risk in these two regions.

## Figures and Tables

**Table 1:** Description of climatic and landscape factors used in this study.

Predictors	Descriptions
Climatic predictors	
Pre_1	Mean precipitation in previous spring
Pre_2	Mean precipitation in previous summer
Pre_3	Mean precipitation in previous autumn
Pre_4	Mean precipitation in previous winter
MeanTem_1	Mean temperature in previous spring
MeanTem_2	Mean temperature in previous summer
MeanTem_3	Mean temperature in previous autumn
MeanTem_4	Mean temperature in previous winter
MaxTem_1	Mean maximum temperature in previous spring
MaxTem_2	Mean maximum temperature in previous summer
MaxTem_3	Mean maximum temperature in previous autumn
MaxTem_4	Mean maximum temperature in previous winter
Landscape factors	
CA_X ^1^	Total area of a land cover class X
PLAND_X ^1^	Percentage of area of a land cover class X
TE_X ^1^	Total edge length of a land cover X at the region
ED_X ^1^	Edge density of a land cover X at the region
DIST_O	Distance to the origin area of Lyme disease

^1^ X—(21–24, 41, 42, and 43). 21, developed-open space; 22—developed-low intensity space; 23—developed-medium intensity space; 24—developed-high intensity space; 41—deciduous forest; 42—evergreen forest; 43—mixed forest.

**Table 2:** Summary (Mean ± S.D.) and univariate regression results (standardized regression coefficient, b, and *t*) for the predictors correlated with the Lyme cases in the Northeast and Upper Midwest United Sates.

Variables	Upper Midwest			Northeast		
	Mean ± S.D.	b	*t*	Mean ± S.D.	b	*t*
Dist_O	125 ± 157	−1.39	−12.9 ***	296 ± 248	−1.25	−12.5 ***
Climatic predictors ^1^						
Pre_1	100 ± 35.4	−0.073	−1.0	90.6 ± 29.3	−0.056	−1.15
Pre_2	82.2 ± 24.5	0.23	3.51 ***	114 ± 38.7	−0.006	−0.095
Pre_3	72.0 ± 29.6	−0.072	−1.02	87.0 ± 26.7	−0.098	−2.09 *
Pre_4	54.9 ± 23.4	−0.56	−5.62 ***	85.9 ± 18.7	0.067	1.13
MeanTem_1	13.4 ± 5.41	−0.066	−1.07	13.2 ± 5.50	0.024	0.52
MeanTem_2	24.4 ± 3.43	−0.15	−2.56 *	24.3 ± 3.76	0.076	1.67
MeanTem_3	13.9 ± 4.36	−0.039	−0.66	13.8 ± 4.36	0.003	0.07
MeanTem_4	0.39 ± 6.99	0.008	0.13	2.41 ± 6.83	−0.016	−0.36
MaxTem_1	15.1 ± 4.02	−1.21	−8.54 ***	17.4 ± 3.88	0.58	4.12 ***
MaxTem_2	28.6 ± 2.07	−0.74	−6.47 ***	28.6 ± 2.39	0.66	6.61 ***
MaxTem_3	16.40 ± 2.29	−0.82	−7.62 ***	18.3 ± 3.02	0.44	3.73 ***
MaxTem_4	2.55 ± 4.67	−1.33	−9.32 ***	6.25 ± 5.18	0.041	0.29
Landscape predictors ^2^						
CA_21	7180 ± 4421	0.874	18.2 ***	1.0E5 ± 7205	0.70	17.2 ***
PLAND_21	4.51 ± 2.75	0.68	14.2 ***	7.14 ± 5.22	0.51	11.9 ***
TE_21	4.6E6 ± 2.3E6	0.95	18.3 ***	6.1E6 ± 3.9E6	0.82	17.8 ***
ED_21	28.9 ± 13.2	0.72	15.0 ***	42.2 ± 23.1	0.56	12.8 ***
CA_22	4786 ± 6960	0.76	17.6 ***	5206 ± 5775	0.69	18.1 ***
PLAND22	3.21 ± 4.59	0.68	15.8 ***	4.12 ± 4.97	0.56	13.1 ***
TE_22	3.1E6 ±3.3E6	0.76	17.7 ***	3.7E6 ± 3.8E6	0.73	18.5 ***
ED_22	20.6 ± 22.7	0.70	16.0 ***	28.4 ± 29.9	0.60	13.9 ***
CA_23	1887 ± 4573	0.84	19.2 ***	2655 ± 4094	0.67	16.9 ***
PLAND_23	1.30 ± 0.64	0.67	14.9 ***	2.51 ± 4.88	0.43	10.0 ***
TE_23	1.3E6 ± 2.5E6	0.77	17.8 ***	1.7E6 ± 2.4E6	0.70	17.5 ***
ED_23	8.84 ± 17.1	0.66	15.1 ***	15.6 ± 26.1	0.49	11.2 ***
CA_24	759 ± 2354	0.88	19.8 ***	998 ± 1729	0.52	12.8 ***
PLAND_24	0.52 ± 1.37	0.64	14.1 ***	1.24 ± 4.28	0.16	3.62 ***
TE_24	3.6E5 ± 9.0E5	0.83	18.9 ***	5.1E5 ± 7.8E5	0.59	14.3 ***
ED_24	2.49 ± 5.80	0.65	14.5 ***	5.44 ± 13.5	0.28	6.32 ***
CA_41	3.2E5 ± 3.2E5	−0.05	−0.64	4.8E5 ± 4.9E5	0.03	0.40
PLAND_41	17.7 ± 13.9	−0.19	−2.91 **	26.3 ± 18.7	0.094	1.66
TE_41	6.8E6 ± 6.1E6	0.19	1.96	9.7E6 ± 8.5E6	0.32	3.46 ***
ED_41	37.0 ± 19.7	0.053	0.77	55.1 ± 27.7	0.22	4.24 ***
CA_42	3293 ± 1.0E5	−0.26	−3.85 ***	1.7E5 ± 3.5E5	−0.75	−7.27 ***
PLAND_42	1.21 ± 2.58	−0.19	−2.95 **	8.36 ± 8.81	−0.57	−9.74 ***
TE_42	1.3E6 ± 3.8E6	−0.23	−3.43 ***	5.5E6 ± 9.4E6	−0.78	−6.45 ***
ED_42	4.98 ± 9.60	−0.14	−2.16 *	27.7 ± 23.1	−0.47	−8.02 ***
CA_43	9616 ± 1.9E5	−0.19	−2.61 **	2.6E5 ± 4.7E5	−0.52	−4.41 ***
PLAND_43	4.11 ± 5.37	−0.18	−2.71 **	13.2 ± 9.62	−0.097	−1.67
TE_43	4.3E6 ± 7.2E6	−0.16	−1.97 *	1.0E7 ± 1.4E7	−0.22	−1.70
ED_43	19.5 ± 20.4	−0.12	−1.78	54.4 ± 31.9	0.002	0.044

Note: * *p* < 0.05; ** *p* < 0.01, *** *p* < 0.001; ^1^ Pre_X, seasonal mean precipitation in previous year, X—(1, spring; 2, summer; 3, autumn; 4, winter); MeanTem_X, mean temperature in pervious year; MaxTem, mean maximum temperature in previous year; ^2^ CA_X, Total area of a land cover class X, X—(21, developed-open space; 22, developed-low intensity space; 23, developed-medium intensity space; 24, developed-high intensity space; 41, deciduous forest; 42, evergreen forest; 43, mixed forest); PLAND_X, Percentage of area of a land cover class X; TE_X, Total edge length of a land cover X; ED_X, Edge density of a land cover X.

**Table 3:** Summary statistics (averaged regression coefficient, b, Z-statistics, and p-values) for the predictors correlated with the Lyme cases in model averaging in the Northeast and Upper Midwest United Sates. For explanation of the variables, see Table 1.

Variables	Upper Midwest			Northeast		
	b	Z	*p*-Value	b	Z	*p*-Value
Dist_O	−1.12	12.8 ***	<0.001	−0.60	5.02 ***	<0.001
Climatic predictors ^1^						
PRE_2	−0.004	0.22	0.827			
PRE_3				−0.003	0.21	0.831
MeanTem_2	0.02	0.565	0.827			
MaxTem_2				0.14	2.07 *	0.038
MaxTem_4	0.001	0.073	0.942			
Landscape predictors ^2^						
TE_21	0.42	4.66 ***	<0.001			
ED_21	0.36	5.09 ***	<0.001			
PLAND_22				−0.022	0.471	0.637
TE_22				0.72	11.76 ***	<0.001
CA_24	0.05	1.10	0.270			
PLAND_24				0.16	4.85 ***	<0.001
PLAND_41	0.34	6.65 ***	<0.001			
TE_41				0.060	0.469	0.638
ED_41				0.30	4.09 ***	<0.001
CA_42	−0.002	0.118	0.906	0.036	0.34	0.731
PLAND_42				−0.22	3.03 **	0.002
PLAND_43	0.003	0.165	0.869			

Note: * *p* < 0.05; ** *p* < 0.01, *** *p* < 0.001. ^1^ Pre_X, seasonal mean precipitation in previous year, X—(2, summer; 3, autumn; 4, winter]; MeanTem_X, mean temperature in pervious year; MaxTem, mean maximum temperature in previous year. ^2^ CA_X, Total area of a land cover class X, X—(21, developed-open space; 22, developed-low intensity space; 24, developed-high intensity space; 41, deciduous forest; 42, evergreen forest; 43, mixed forest); PLAND_X, Percentage of area of a land cover class X; TE_X, Total edge length of a land cover X; ED_X, Edge density of a land cover X.
